# Which Neuropsychological Tests? Predicting Cognitive Decline and Dementia in Parkinson’s Disease in the ICICLE-PD Cohort

**DOI:** 10.3233/JPD-212581

**Published:** 2021-08-02

**Authors:** Rachael A. Lawson, Caroline H. Williams-Gray, Marta Camacho, Gordon W. Duncan, Tien K. Khoo, David P. Breen, Roger A. Barker, Lynn Rochester, David J. Burn, Alison J. Yarnall, on behalf of the ICICLE-PD study group

**Affiliations:** a Translational and Clinical Research Institute, Newcastle University, UK; b Department of Clinical Neurosciences, University of Cambridge, UK; c Centre for Clinical Brain Sciences, University of Edinburgh, UK; d NHS Lothian, Edinburgh, UK; e School of Medicine and Menzies Health Institute Queensland, Griffith University, Australia; f School of Medicine, University of Wollongong, Australia; g Anne Rowling Regenerative Neurology Clinic, University of Edinburgh, UK; h Usher Institute of Population Health Sciences and Informatics, University of Edinburgh, UK; i Wellcome MRC Cambridge Stem Cell Institute, University of Cambridge, UK; j Newcastle Upon Tyne Hospitals NHS Foundation Trust, UK; k Faculty of Medical Science, Newcastle University, UK

**Keywords:** Parkinson’s disease, neurocognitive disorders, cognitive dysfunction, neuropsychological tests

## Abstract

**Background:**

Cognitive impairment is common in Parkinson’s disease (PD), with 80% cumulatively developing dementia (PDD).

**Objective:**

We sought to identify tests that are sensitive to change over time above normal ageing so as to refine the neuropsychological tests predictive of PDD.

**Methods:**

Participants with newly diagnosed PD (*n* = 211) and age-matched controls (*n* = 99) completed a range of clinical and neuropsychological tests as part of the ICICLE-PD study at 18-month intervals over 72 months. Impairments on tests were determined using control means (<1-2SD) and median scores. Mild cognitive impairment (PD-MCI) was classified using 1-2SD below normative values. Linear mixed effects modelling assessed cognitive decline, while Cox regression identified baseline predictors of PDD.

**Results:**

At 72 months, 46 (cumulative probability 33.9%) participants had developed PDD; these participants declined at a faster rate in tests of global cognition, verbal fluency, memory and attention (*p* < 0.05) compared to those who remained dementia-free. Impaired baseline global cognition, visual memory and attention using median cut-offs were the best predictors of early PDD (area under the curve [AUC] = 0.88, *p* < 0.001) compared to control-generated cut-offs (AUC = 0.76–0.84,

*p* < 0.001) and PD-MCI (AUC = 0.64–0.81, *p* < 0.001). Impaired global cognition and semantic fluency were the most useful brief tests employable in a clinical setting (AUC = 0.79, *p* < 0.001).

**Conclusion:**

Verbal fluency, attention and memory were sensitive to change in early PDD and may be suitable tests to measure therapeutic response in future interventions. Impaired global cognition, attention and visual memory were the most accurate predictors for developing a PDD. Future studies could consider adopting these tests for patient clinical trial stratification.

## INTRODUCTION

Cognitive impairment is a frequent non-motor complication of Parkinson’s disease (PD), with up to 80% of patients developing dementia (PDD) 20 years after disease onset [1]. Currently, there are no effective treatments which slow cognitive decline in PD [2, 3]. A limiting factor in the development of interventions has been the identification of appropriate outcome measures that are sensitive to change due to medication response compared with normal aging. Commonly used neuropsychological tests may not be sensitive to change over time [4], and therefore not ideal cognitive measures for identifying clinically meaningful improvements due to pharmacological or non-pharmacological interventions [5].

Cognitive impairment in PDD is heterogeneous and multiple cognitive domains are involved [1, 6]; impairment in global cognition [7], executive function [8, 9], semantic fluency and visuospatial function [10], attention [9, 11] and memory [12, 13] have all been associated with the development of PDD. However, a number of these studies did not incorporate an age-matched control group to generate age-appropriate normative data [10, 14–16], included heterogeneous cohort of participants with different disease durations, small sample sizes [9, 17], or only used a limited battery of neuropsychological tests [7, 10, 13, 18, 19]. While it is now clear that patients fulfilling PD-MCI criteria are associated with an increased risk of developing future PDD [11, 14, 20–22]; from a clinical and research perspective it would be more useful to know which cognitive deficits and tests are most predictive of developing an early PDD. Understanding early impairments in specific neuropsychological tests and identifying optimal cut-offs that predict the early development of dementia is vital for future care planning and recruiting participants to clinical trials of potentially disease modifying therapies [4].

The aims of this prospective longitudinal study were firstly, to determine which cognitive tests were sensitive to changes, over and above normal aging, in those with early PD; and secondly, identify those cognitive tests that are most sensitive in predicting which participants will subsequently develop an early PDD. We hypothesised that neuropsychological tests measuring attention [11], semantic fluency and visuospatial function [10] would be associated with faster rates of cognitive decline in PD and the development of PDD, independent of normal aging.

## METHODS

### Participants

Between June 2009 and December 2011, newly diagnosed PD patients from the community and hospital outpatient clinics in Newcastle-upon-Tyne, Gateshead and Cambridgeshire, UK were invited to participate in the Incidence of Cognitive Impairments in Cohorts with Longitudinal Evaluation in Parkinson’s disease (ICICLE-PD) study (*n* = 211) [23]. Participants were re-assessed at 18-month intervals for up to 72 months. Idiopathic PD was diagnosed by a movement disorder specialist and fulfilled Queen’s Square Brain Bank criteria [24]. Healthy control subjects were recruited from the community to provide age, sex and culturally appropriate normative data (*n* = 99). Full exclusion criteria have been published elsewhere [23]. Briefly, participants were excluded if they had significant cognitive impairment at presentation (Mini-Mental State Examination (MMSE) < 24) or a pre-existing diagnosis of dementia [6], an atypical parkinsonian syndrome, or insufficient English to complete assessments.

This study was approved by the Newcastle and North Tyneside Research Ethics Committee. All subjects provided written informed consent.

### Assessments

Demographic information including age, gender and years of education was collected. Participants were assessed using the Movement Disorder Society (MDS) Unified Parkinson’s Disease Rating Scale (MDS-UPDRS) part III [25], Hoehn and Yahr staging and Geriatric Depression Scale (GDS-15) [26]. Participants were assessed in an “on” motor state; levodopa equivalent daily dose (LEDD) was calculated [27].

Participants completed a schedule of neuropsychological tests (Supplementary Table 1). Global cognitive function was assessed using the MMSE [28] and Montreal Cognitive Assessment (MoCA) [29]; the naming and sentence items from the MoCA were used to assess language. Executive function was assessed using the One Touch Stockings from the Cambridge Neuropsychological Test Automated Battery (CANTAB) [30], phonemic fluency (number of words generated in 60 s beginning with the letter F) and semantic fluency (number of animals generated in 90 s). Visuospatial function was evaluated using the pentagon copying item within the MMSE and graded using a modified 0–2 rating scale [31]. Memory was assessed using the Pattern Recognition Memory (PRM), Spatial Recognition Memory (SRM) and Paired Associate Learning (PAL) tests from CANTAB [30]. Attention was assessed using the Cognitive Drug Research (CDR) battery [32]: Simple Reaction Time (SRT), Choice Reaction Time (CRT) and Digit Vigilance (DV). Spatial working memory was assessed using the Spatial Working Memory test (SWM) from the CDR battery.

We identified mild cognitive impairment (PD-MCI) [33] as previously described by Yarnall et al. [23]. Participants were classified as PD-MCI if they reported subjective cognitive decline and performed 1, 1.5 or 2 standard deviations (SD) or more below the mean of appropriate norms (controls) on at least two neuropsychological tests across five cognitive domains: attention (PoA and digit vigilance accuracy), memory (PRM number correct, SRM number correct and PAL mean trials to success), executive function (OTS number solved on first choice, semantic and phonemic fluency), language (naming and sentence subsets from the MoCA) and visuospatial function (pentagon copying). In addition, PD-MCI was defined as an impaired MoCA (< 26) plus subjective cognitive decline. Subjective cognitive decline and functional independence were determined through semi-structured interviews with participants and/or their carers to enable a diagnosis of PDD to be made using the MDS criteria [6].

The medical notes of all participants who did not return for 72-month follow-up were also reviewed to capture any additional diagnosis of dementia. A diagnosis was made by expert consensus (Newcastle: AJY and RAL, Cambridge: CHWG and MC); date of diagnosis was recorded as the midpoint between research or clinic visits.

### Statistical analysis

Statistical Analysis was conducted using SPSS (IBM Corp. V.24, USA) and R software (Version 3.4.0; R Foundation for Statistical Computing, Vienna, Austria). Data were examined for normality of distribution with visual histograms and Kolmogorov-Smirnov’s test. Comparisons of means between two groups were performed using independent *t*-tests or Mann-Whitney U tests as appropriate. Ordinal data were compared using chi-squared tests. Survival and cumulative survival were calculated using Kaplan-Meier plots.

Within R, *lme4* was used to perform linear mixed effects modelling (LMEM) to determine change in cognitive measures from baseline to 72 months in PD vs. controls and in PD vs. PDD participants (see Supplementary Methods for details). Due to the longitudinal nature of this study, there were some missing data (Supplementary Table 2); LMEM is suitable for longitudinal data analysis because of its ability to handle missing data. Backwards stepwise Cox regression identified baseline predictors of PDD using a data driven approach (Supplementary Methods). Cognitive scores were dichotomised as impaired using: i) cut-offs at 1SD, 1.5SD and 2SD below control mean scores, and ii) using median scores (Supplementary Table 3). These cut-offs were used as 1, 1.5 and 2SDs below normative values and have been commonly applied in the literature, while a median cut-off is typically used in cohorts without a control group or where normative data are not available. An additional model using impaired median scores and pen and paper only tests (MoCA, MMSE, semantic fluency, phonemic fluency and pentagons) was performed to identify tests which may be useful in an outpatient setting. Finally, baseline PD-MCI classifications versus no cognitive impairment, using impaired MoCA (< 26) and 1SD (≤1SD but > 1.5SD), 1.5SD (≤1.5SD but > 2SD) and 2 SD (≤2SD) cut-offs were individually added to the basic model. Model fit was assessed using log likelihood ratios, and area under the curve (AUC) was calculated for each model using receiver operating characteristic (ROC) curves. For all analyses, we applied Benjamini-Hochberg multiple comparisons correction with a 5% false discovery rate.

### Data availability statement

Unidentifiable data may be shared on request.

## RESULTS

**Fig. 1:**
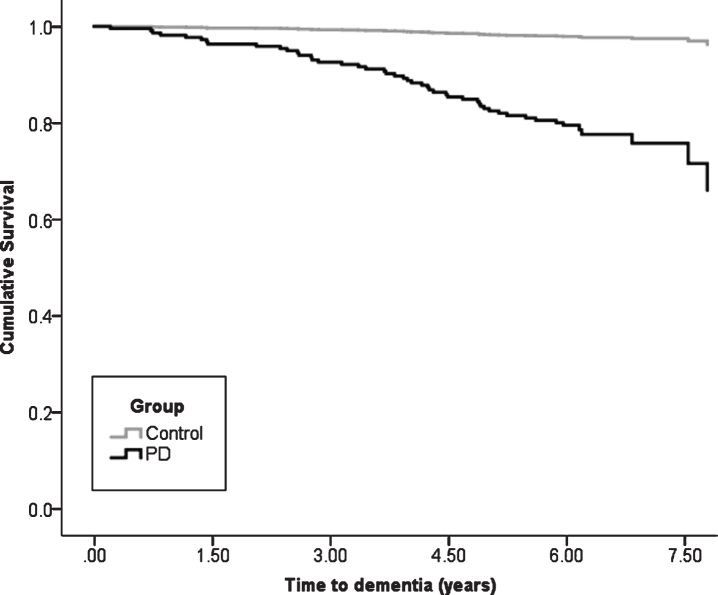
Kaplan-Meier plot of time to dementia diagnosis in Parkinson’s vs. control participants. PD, Parkinson’s disease.

At baseline, after exclusions, 211 participants with PD with a mean disease duration of 5.6±5.1 months (Table 1) and 99 healthy controls completed assessments (Supplementary Table 1). Over 72 months, 106 (50.2%) PD participants and 66 (66.7%) controls returned for in person evaluation. Mean duration of follow-up was 4.3±2.2 years (median = 6.0 IQR = 3.0 years). Of the 211 PD participants, 46 (cumulative dementia probability of 33.9%) developed PDD by the end of the 72-month assessment period, compared to two (cumulative dementia probability of 2.4%) controls who both were diagnosed with Alzheimer’s disease dementia (*χ*^2^ = 17.0, *p* < 0.001, Fig. 1). Mean time to dementia diagnosis was 3.8±1.8 years in PD participants who reached this outcome and 5.5±0.6 years in controls.

**Table 1:** Baseline demographic and clinical characteristics of cohort

Variable	Control (*n* = 99)		PD (*n* = 211)		t/Z	*p*
	Mean	SD	Mean	SD
Age (y)	67.9	8.2	65.9	9.8	1.8	0.690
Education (y)	13.1	3.4	12.8	3.6	–0.9	0.381
NART	115.9	8.7	114.4	10.3	–0.8	0.434
PD duration (mo)			5.6	5.1
MDS UPDRS III total			27.4	11.8
Hoehn &Yahr stage			1.9	0.7
LEDD mg/day			176.9	154.7
GDS-15	1.0	1.5	2.8	2.6	–7.2	**< 0.001**
MoCA total	27.0	2.5	25.4	3.4	–3.7	**< 0.001**
MMSE total	29.0	1.2	28.7	1.3	–2.5	**0.014**
Verbal fluency (F)	13.0	4.7	11.9	4.8	–2.0	**0.047**
Semantic fluency (Animals)	23.9	6.1	21.3	6.5	3.3	**0.001**
OTS no. problems solved 1st choice	16.4	2.5	14.6	4.0	–4.0	**< 0.001**
PRM number correct	20.7	2.5	19.7	3.2	–2.8	**0.005**
PRM % correct	86.3	10.2	81.2	13.8	–2.9	**0.004**
SRM % correct	80.7	9.2	76.0	11.6	–3.0	**0.003**
SRM number correct	16.1	1.8	15.3	2.2	–2.9	**0.004**
PAL stages completed	7.8	0.6	7.1	1.2	–5.5	**< 0.001**
PAL total errors	19.2	14.5	21.3	16.5	–1.0	0.313
PAL total trials	14.1	3.7	14.8	4.4	–1.0	0.297
PAL mean trials to success	1.8	0.6	2.0	0.8	–1.7	0.090
SRT mean (ms)	315.3	63.0	361.7	125.8	–4.7	**< 0.001**
DV accuracy %	96.0	5.8	92.2	12.9	–1.9	0.057
DV mean (ms)	452.0	44.8	476.9	56.8	–3.6	**< 0.001**
CRT accuracy %	97.0	2.7	97.2	2.5	–0.6	0.574
CRT mean (ms)	510.6	60.3	540.8	94.5	–2.4	**0.018**
PoA (ms)	1277.9	136.0	1379.3	235.4	–3.9	**< 0.001**
PoA CoV	50.2	10.2	54.0	11.7	–2.5	**0.014**
Continuity of Attention	91.7	3.5	90.1	6.2	–1.6	0.112
Cognitive Reaction Time	195.4	53.6	179.1	98.4	–1.0	0.306
SWM original stim accuracy %	93.5	9.9	90.8	14.6	–0.6	0.538
SWM new stim accuracy %	94.3	12.1	92.4	12.6	–1.9	0.059
SWM SI %	0.9	0.2	0.8	0.2	–1.0	0.318
SWM original stim mean (ms)	1121.0	456.3	1173.3	446.9	–1.0	0.338
SWM new stim mean (ms)	1166.7	331.8	1261.6	495.6	–0.8	0.402
SWM mean (ms)	1152.9	406.4	1221.5	459.8	–0.8	0.398
Pentagon score	1.9	0.2	1.9	0.4	–2.1	**0.036**
Naming score	2.9	0.4	2.9	0.3	–0.9	0.351
Sentence score	1.7	0.6	1.6	0.6	–1.4	0.148
	n	%	n	%	*χ*2	p
Gender: male	54	54.5	113	63.0	2.0	0.154
PD-MCI MoCA			100	47.4
PD-MCI 1SD			138	65.4
PD-MCI 1.5SD			87	41.2
M PD-MCI 2SD			44	20.9

PD, Parkinson’s disease; SD, standard deviation; NART, National Adult Reading Test; MDS-UPDRS III, Movement Disorder Society Unified Parkinson’s Disease Rating Scale; LEDD, Levodopa equivalent daily dose; GDS-15, Geriatric Depression Scale; MoCA, Montreal Cognitive Assessment; MMSE, Mini-Mental State Examination; OTS, One Touch Stockings; PRM, paired recognition memory; SRM, spatial recognition memory; PAL, paired associated learning; CRT, choice reaction time; PoA, power of attention; CoV, coefficient of variance; SWM, spatial working memory; SI, sensitivity indices; PD-MCI, mild cognitive impairment with Parkinson’s disease.

### Cognitive change over time

Linear mixed effects modelling, adjusting for covariates, determined change in neuropsychological tests over time (Table 2) in control vs. PD participants, and in PD participants who remained dementia-free vs. those who developed PDD. After corrections for multiple comparisons, only performance in tests of attention (CRT mean and PoA) were sensitive to decline in the PD participants over and above controls (and thus normal aging), and in those who developed PDD. However, performance in tests across many cognitive domains showed a more rapid decline in those participants who developed early PDD compared to those who did not (Table 2); specifically, global cognition (MoCA and MMSE), semantic and phonemic fluency, memory (PRM and PAL) and spatial working memory (SWM accuracy and speed). Additional measures of attention (digit vigilance accuracy, CRT accuracy, CoV fluctuating attention) declined in those who developed early PDD compared to those who did not (*p* < 0.023 for all). In controls and PD participants who did not develop dementia, phonemic fluency scores significantly improved over time (*p* < 0.023), suggesting a possible learning effect. To account for baseline PD-MCI, this analysis was repeated adjusting for baseline PD-MCI classification; this did not alter the results (Supplementary Table 4).

**Table 2:** Neuropsychological tests results over time in PD vs. controls and PD vs. PDD participants

Cognitive domain	Neuropsychological test	PD vs. control participants^a^						PD vs. PDD participants^b^					
		Time		Group		Time×Group		Time		PDD		Time×PDD	
		β	*p*	β	*p*	β	*p*	β	*p*	β	*p*	β	*p*
Global cognition	MoCA^*^	–0.1	0.312	–1.3	**< 0.001**	0.0	0.960	0.8	**< 0.001**	–2.6	**< 0.001**	–0.7	**< 0.001**
	MMSE^*^	–0.2	0.040	–0.3	0.043	–0.2	0.048	0.4	**0.006**	–0.6	**0.006**	–0.9	**< 0.001**
Executive function and Verbal fluency	Phonemic Fluency^*^	0.2	0.185	–0.6	0.274	0.5	**0.017**	1.6	**< 0.001**	–2.3	**0.006**	–1.1	**0.002**
	Semantic Fluency^*^	–0.3	0.129	–1.9	**0.005**	0.2	0.317	0.8	0.024	–3.5	**< 0.001**	–1.3	**< 0.001**
	*OTS no. solved on first choice* ^†^	0.1	0.992	–2.2	0.315	4.6	0.376	–0.8	0.900	–5.3	0.313	7.1	0.405
Memory	*PRM number correct* ^†^	0.1	0.468	–0.9	**0.007**	–0.1	0.670	0.3	0.138	–1.6	**0.001**	–0.7	**0.004**
	*PRM % correct* ^†^	0.4	0.497	–3.6	**0.009**	–0.3	0.656	1.4	0.153	–6.5	**0.002**	–2.8	**0.004**
	*SRM number correct* ^†^	–0.3	**0.016**	–0.9	**< 0.001**	0.0	0.853	–0.2	0.309	–1.8	**< 0.001**	–0.1	0.564
	*SRM % correct* ^†^	–1.8	**0.014**	–4.4	**0.001**	–0.1	0.884	–1.1	0.298	–8.7	**< 0.001**	–0.5	0.654
	*PAL stages complete* ^†^	0.0	0.839	–0.6	**< 0.001**	0.1	0.098	0.1	0.461	–0.5	**0.006**	–0.1	0.350
	*PAL total errors* ^†^	0.1	0.919	1.4	0.458	0.3	0.770	–1.5	0.302	2.4	0.369	2.6	0.061
	*PAL total trials* ^†^	–0.1	0.616	0.9	0.079	0.6	0.063	–0.3	0.636	–0.1	0.855	1.9	**< 0.001**
	*PAL mean trials to success* ^†^	0.0	0.963	0.2	0.026	0.1	0.093	–0.1	0.460	0.4	**0.004**	0.3	**< 0.001**
Attention	*SRT mean* ^†^	7.6	0.284	25.1	0.027	10.9	0.189	–17.8	0.171	35.1	0.046	17.8	0.145
	*Digit vigilance accuracy* ^†^	0.1	0.932	–3.0	0.023	–1.0	0.198	2.1	0.037	–8.1	**< 0.001**	–4.2	**< 0.001**
	*Digit vigilance mean* ^†^	0.3	0.924	24.8	**< 0.001**	5.3	0.083	–5.8	0.219	39.9	**< 0.001**	–0.6	0.890
	*CRT accuracy* ^†^	0.6	**0.002**	0.2	0.490	–0.5	0.026	0.3	0.361	–0.8	0.090	–0.7	**0.022**
	*CRT Mean* ^†^	10.5	0.087	22.8	0.023	18.4	**0.011**	8.1	0.441	60.6	**< 0.001**	40.6	**< 0.001**
	*PoA* ^†^	19.7	0.137	72.9	**0.001**	35.7	**0.022**	–8.4	0.721	134.1	**< 0.001**	57.5	**0.012**
	*PoA CoV* ^†^	1.5	0.048	2.4	0.064	–0.2	0.796	0.9	0.446	3.0	0.102	4.0	**< 0.001**
	*Continuity of attention* ^†^	0.3	0.282	–1.2	0.051	–0.6	0.078	1.1	**0.020**	–4.1	**< 0.001**	–2.3	**< 0.001**
	*Cognitive reaction time* ^†^	2.3	0.690	–4.5	0.645	4.1	0.531	11.7	0.235	25.8	0.080	12.1	0.189
Spatial working memory	*SWM original accuracy* ^†^	0.3	0.737	–2.9	0.089	–0.7	0.506	0.0	0.989	–6.1	**0.015**	–1.8	0.249
	*SWM new accuracy* ^†^	–0.5	0.676	–3.4	0.037	–2.2	0.084	–3.9	0.046	–2.8	0.241	–4.6	**0.018**
	*SWM SI* ^†^	0.0	0.939	–0.1	0.036	0.0	0.261	0.0	0.319	–0.1	0.051	–0.1	0.051
	*SWM original speed* ^†^	10.9	0.806	68.1	0.275	68.1	0.195	–1.7	0.983	312.0	**< 0.001**	254.5	**0.002**
	*SWM new speed* ^†^	22.0	0.597	110.6	0.082	58.5	0.232	35.2	0.626	309.5	**0.001**	235.7	**0.001**
	*SWM mean speed* ^†^	14.5	0.738	85.0	0.162	74.6	0.146	20.3	0.792	302.5	**< 0.001**	264.4	**0.001**

Significant results after Benjamini–Hochberg procedure are highlighted in bold p < 0.023. ^a^Covariates included in the model: age, education, sex and GDS-15. ^b^Covariates included in the model: age, MDS-UPDRS III, Sex, GDS-15, Education, Time x MDS-UPDRS III. ^*^Time points included: baseline, 18, 36, 54 and 72 months; ^†^Time points included: baseline, 18, 36 and 54 months. Full details of missing data is available in Supplementary Table 2. MoCA, Montreal Cognitive Assessment; MMSE, Mini-Mental State Examination; OTS, One Touch Stockings; PRM, paired recognition memory; SRM, spatial recognition memory; PAL, paired associated learning; CRT, choice reaction time; PoA, power of attention; CoV, coefficient of variance; SWM, spatial working memory; SI, Sensitivity indices; MDS-UPDRS III, Movement Disorder Society Unified Parkinson’s Disease Rating Scale; GDS-15, Geriatric Depression Scale.

### Baseline predictors of PDD

Backwards Cox regression identified that baseline median age (> 66 years, HR = 3.4, *p* < 0.001) and median MDS-UPDRS III score (> 26, HR = 2.7, *p* < 0.01) were significantly predictive of developing PDD earlier and were therefore included in each model predicting PDD progression (Table 2). Impaired global cognition (MoCA) and attention (PoA) at baseline were significant predictors of developing an early PDD in every model (HR = 3.2–4.9 and HR = 2.3–3.9, respectively), irrespective of the cut-off applied. Impaired attention as measured by digit vigilance accuracy was a significant predictor in the 1SD, 2SD and median scores models (HR = 2.4–4.9), but not 1.5SD. Impaired memory was also a predictor in each model, but the specific tests varied, with SRM (HR = 2.1 and HR = 3.1, respectively) significant in the 1SD and 1.5SD cut-off models, impaired PRM (HR = 5.0) using the 2SD cut-off, and impaired PAL (HR = 3.4) using the median cut-off. Impaired visuospatial function, based on pentagon copying, was only a significant predictor in the 2SD model (HR = 12.2). Using the pen and paper tests only, impaired MoCA (< 26, HR = 5.7) and semantic fluency (< 21, HR = 2.9) were significant predictors of PDD.

**Fig. 2:**
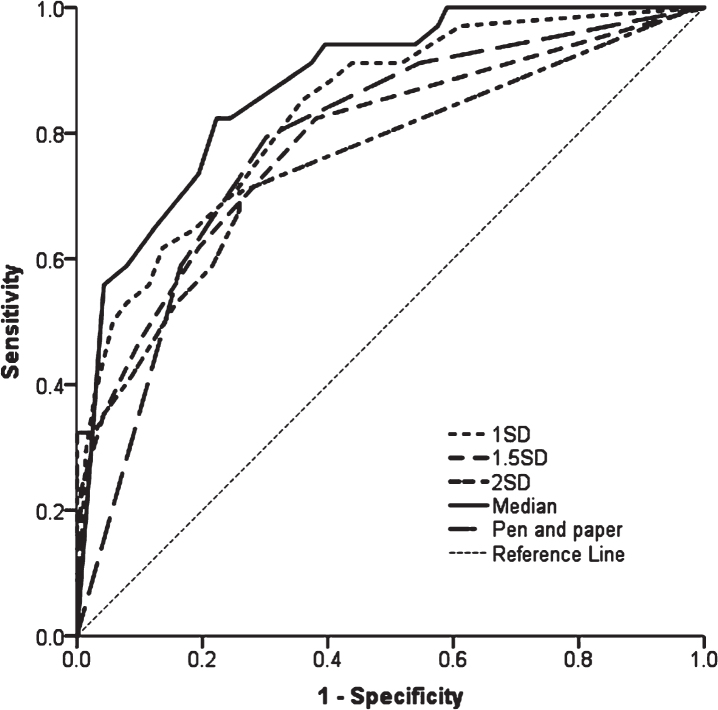
ROC curves of models predicting PDD using baseline cognitive tests. 1SD = model using 1 SD cut-offs below controls, cognitive tests included MoCA, SRM, DV accuracy and PoA; 1.5SD = model using 1.5 SD cut-offs below controls, cognitive tests included MoCA, SRM and PoA; 2SD = model using 2 SD cut-offs below controls, cognitive tests included MoCA, PRM, DV accuracy, PoA and pentagons; Median = model using median scores as cut-off, cognitive tests included MoCA, PAL, DV accuracy and PoA; Pen and paper = model using median scores as cut-offs for MoCA and semantic fluency. ROC, Receiver operating Characteristic; PDD, Parkinson’s disease with dementia; SD, standard deviation; MoCA, Montreal Cognitive Assessment; SRM, spatial recognition memory; PRM, paired recognition memory; DV, Digit Vigilance; PAL, paired associated learning; PoA, power of attention.

Comparing the models (Fig. 2), AUC ranged from 0.758–0.876 (*p* < 0.001 for all, Table 2). The model using median scores of neuropsychological tests was the best fit, demonstrated by the lowest log-likelihood (267.6, *p* < 0.001) and highest AUC (AUC = 0.876, *p* < 0.001) compared to other models.

### Baseline PD-MCI and conversion to PDD

**Fig. 3:**
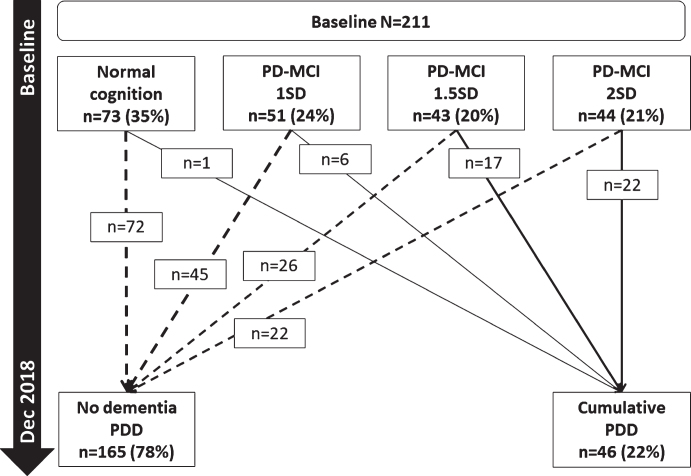
Baseline PD-MCI and progression to PDD. PD-MCI, Parkinson’s disease with mild cognitive impairment; SD, standard deviation; PDD, Parkinson’s disease dementia.

Of the 46 participants who developed PDD, 45 (97.8%, Fig. 3) had PD-MCI at baseline (using < 1-2SD criteria). Most participants with normal cognition at baseline (*n* = 72, 98.6%) remained dementia-free over 72 months. Of the participants who met PD-MCI criteria at baseline, the numbers developing PDD within 72 months were: six (11.8%) of those meeting PD-MCI 1SD criteria (≤1SD below controls but > 1.5SD), 17 (39.5%) of those meeting PD-MCI 1.5SD criteria (≤1.5SD below controls but > 2SD); and 22 (50.0%) of those meeting PD-MCI 2SD criteria.

Cox regression modelling with each baseline PD-MCI classification and covariates found that PD-MCI defined by impaired MoCA or 1.5SD and 2SD cut-offs significantly predicted the development of PDD (*p* < 0.01 for all, Table 3), but not PD-MCI defined by the 1SD cut-off (*p* > 0.05). However, PD-MCI 2SD had the highest hazard ratio (HR = 21.9, *p* < 0.001), the best model fit (Table 3, *p* < 0.001) and greatest AUC (AUC = 0.814, *p* < 0.001). Comparing all models, the model fits using PD-MCI criteria had lower predictive power compared to the model of impaired median global cognition, memory, and attention (AUC = 0.876, *p* < 0.001, Table 3).

**Table 3:** Cox regression models of baseline predictors of PDD

Model	Variables in model	β	SE	*p*	HR	HR 95.0% CI		Model fit				AUC 95.0% CI	
						Lower	Upper	LL	*χ*2	*p*	AUC	Lower	Upper
*1SD cut-offs*								292.0	60.4	**< 0.001**	0.835	0.761	0.910
	*MoCA*	1.6	0.4	**< 0.001**	4.9	2.3	10.4
	*SRM No. correct*	0.7	0.4	**0.044**	2.1	1.0	4.2
	*DV accuracy*	1.0	0.4	**0.008**	2.7	1.3	5.6
	*PoA*	0.8	0.4	**0.030**	2.3	1.1	5.0
*1.5SD cut-offs*								303.9	54.3	**< 0.001**	0.785	0.693	0.877
	*MoCA*	1.2	0.4	**< 0.001**	3.3	1.6	6.7
	*SRM No. Correct*	1.1	0.4	**< 0.001**	3.1	1.5	6.1
	*PoA*	1.1	0.3	**0.002**	3.0	1.5	5.9
*2SD cut-offs*								305.4	59.7	**< 0.001**	0.758	0.658	0.858
	*MoCA*	1.2	0.4	**0.002**	3.2	1.5	6.5
	*PRM No. correct*	1.6	0.4	**< 0.001**	5.0	2.2	11.2
	*DV Accuracy*	0.9	0.4	**0.031**	2.4	1.1	5.2
	*PoA*	1.1	0.4	**0.004**	3.0	1.4	6.3
	*Pentagons*	2.5	1.1	**0.026**	12.2	1.3	111.7
*Median cut-offs*								267.6	60.1	**< 0.001**	0.876	0.815	0.936
	*MoCA*	1.6	0.4	**< 0.001**	4.7	2.0	11.1
	*PAL mean trials to success*	1.2	0.5	**0.008**	3.4	1.4	8.4
	*DV accuracy*	1.6	0.5	**0.001**	4.9	1.9	13.1
	*PoA*	1.4	0.5	**0.006**	3.9	1.5	10.5
*Median cut-offs: pen and paper*								348.6	40.9	**< 0.001**	0.785	0.701	0.869
	*MoCA*	1.7	0.4	**< 0.001**	5.7	2.6	12.5
	*Semantic fluency*	1.0	0.4	**0.005**	2.9	1.4	5.9
	*PD-MCI MoCA*	1.7	0.4	**< 0.001**	5.4	2.5	11.6	4.17.8	43.3	**< 0.001**	0.725	0.646	0.804
	*PD-MCI*≤*1SD to 1.5SD*	1.2	0.7	0.096	3.3	0.8	13.1	71.8	3.1	0.078	0.638	0.451	0.824
	*PD-MCI*≤*1.5SD to 2SD*	2.3	0.8	**0.002**	10.3	2.3	47.0	141.1	29.6	**< 0.001**	0.782	0.672	0.892
	*PD-MCI*≤*2SD*	3.1	0.7	**< 0.001**	21.9	5.1	94.4	173.0	44.6	**< 0.001**	0.814	0.720	0.907

Significant results after Benjamini–Hochberg procedure are highlighted in bold p < 0.074. Covariates in each model: median age and median MDS-UPDRS part III score. HR, hazard ratio; CI, confidence interval; LL, Log likelihood; AUC, area under the curve; SD, standard deviation; MoCA, Montreal Cognitive Assessment; SRM, spatial recognition memory; PRM, paired recognition memory, DV, Digit Vigilance, PAL, paired associated learning; PoA, power of attention; MDS-UPDRS III, Movement Disorder Society Unified Parkinson’s Disease Rating Scale part III.

## DISCUSSION

We sought to identify tests that are sensitive to change over time in those with PD above normal ageing and refine the neuropsychological tests predictive of developing an early PDD. In our cohort, the cumulative probability of PDD was 34% within six years compared to 2% of controls who developed dementia. This is comparable to conversion rates reported by previous studies of 17–27% over 4–7 years after PD diagnosis [35–37]. This is the first study to demonstrate that performance in tests of attention are sensitive to changes over time in individuals with newly diagnosed PD, independent of normal ageing, whereas deficits on tests of global cognition, memory, verbal fluency and spatial working memory in addition to attention are more sensitive to cognitive decline in those who develop an early PDD. We propose that performance in these tests may be suitable outcome measures of therapeutic response in clinical trials, as well as informing clinicians planning for the future medical needs for patients. We found that identifying PD-MCI at baseline, while predicting future PDD, does so with much less accuracy than impairments in specific tests. In keeping with previous studies, baseline impaired global cognitive function [7], attention [11] and visual memory [12] consistently predicts future PDD across a range of cut-offs, and therefore, may have utility for patient stratification in clinical trials. In addition, we found that impaired MoCA and semantic fluency scores were predictive of early dementia and could be useful screening tools for routine clinical practice.

A paucity of studies has explored which neuropsychological tests are sensitive to change over time in PD above that seen with normal aging, in comparison to studies that have sought to identify baseline predictors of an early PDD. We found that participants with PD and PDD declined in attention scores at a faster rate over time compared to normal ageing, but only tests of global cognition, memory, verbal fluency and spatial working memory were sensitive to cognitive decline in those who develop PDD. A number of previous studies have shown associations between PDD and poorer performance on a range of global and specific cognitive domains (including attention, memory and executive function [7, 8, 10–12]), although many are cross-sectional. A previous study in PD, albeit in a smaller sample size (*n* = 59 PD participants vs. *n* = 40 controls over 5 years), reported that the performance of PD participants significantly declined at a faster rate in tests of psychomotor speed, memory, executive function and visuospatial function compared to controls, although associations with subsequent dementia were not assessed [17]. A retrospective study in PD (*n* = 118 over 5 years) reported that participants who subsequently developed PDD showed a significantly faster rate of decline in performance in visuospatial and verbal memory tests compared to those with AD, and that cognitive decline preceded the diagnosis of dementia by several years [13]. In advanced dementia-free PD (PD duration≥10 years, *n* = 49), worsening attention and executive function have been associated with development of PDD [9]. Furthermore, impaired attention has been associated with Lewy body pathologies [38, 39]. In participants with a REM sleep behavioural disorder, impaired test performance in measures of attention and executive function were observed six years prior to a dementia with Lewy body diagnosis [40]. These findings support our results, which suggests that the performance on selective tests of global cognition, attention, memory, verbal fluency, and spatial working memory we identified in this study are sensitive to change over time in early PDD, and so could be considered as outcome measures in future clinical trials investigating interventions targeting cognitive decline or for inclusion in trials of disease modifying agents.

This is the first study to explore a range of cut-offs of neuropsychological tests using normative data. We identified that baseline impaired global cognition, attention and memory consistently predict the development of future PDD across a range of cut-offs, specifically, impaired MoCA and PoA were significant predictors in every model; digit vigilance accuracy was impaired in all but one model (1.5SD below controls), with variations of impaired visual memory across models (SRM, PRM or PAL). Consistent with the CamPaIGN study [10], pentagon copying was predictive of early PDD in addition to impaired global cognition, attention and memory, but only in the 2SD model (< 1 for 2SD compared to < 2 for all other cut-offs). This may be due to the limited scoring of this measure (0, 1 or 2) which may be insensitive to subtle visuospatial dysfunction. Contrary to our hypothesis, models using median cut-offs had the strongest predictive power compared to cut-offs generated using normative data. A strength of this study is the inclusion of age-matched controls to provide normative data. However, on inspection of the cut-offs (Supplementary Table 3), those calculated using normative data (1-2SD below controls) had greater degrees of impairment compared to those using the median values. This suggests that using stricter age-generated cut-offs may have not detected more subtle cognitive impairments. Previous studies have used a range of neuropsychological tests and from these it is unclear which tests should be used in research and clinical settings. A previous longitudinal study reported that the development of PDD over a mean of 5 years was associated with verbal, visuospatial and working memory deficits [13]. However, cut-offs used to identify impairment were not explored or identified. We propose that MoCA, PoA, digit vigilance accuracy and PAL could be utilised to identify those at risk of developing PDD in the future.

Using non-computerised tests that are commonly used in clinic, we found that impaired MoCA and semantic fluency significantly predicted PDD. The CamPaIGN study [10] reported that impaired baseline semantic fluency and pentagon copying were predictors of developing PDD within ten years of PD diagnosis, with another study also reporting that semantic fluency and figure copying was predictive of PDD [15]. However, impaired pentagon copying as a predictor of future PDD was only seen using the 2SD cut-off (scores < 1) in the present study, but not using other cut-off scores (< 2). We found that impaired performance on the MoCA (< 26), a brief measure of global cognitive function - which includes a cube-copying task (a visuospatial function test similar to the pentagon-copying task) - was a predictor of subsequent PDD. A recent study comparing three cognitive screening measures found that the MoCA was the only measure associated with development of PDD and a faster rate of progression to dementia [7]. This may be due in part to a greater weight of tests of attention, visuospatial and executive function tests in the MoCA compared to the MMSE [41, 42]. Nonetheless, impaired MoCA and semantic fluency (90 s) had high accuracy with an AUC of 0.79, and this model was comparable to—or had a better fit than—models containing more comprehensive tests or PD-MCI. This suggests that these two commonly used tests may be suitable in routine clinical practice to identify patients likely to develop an early PDD, using cut-off scores of 26 and 21, respectively.

We showed that the presence of baseline PD-MCI using a 1.5SD or 2SD cut-off significantly increased the risk of developing PDD within six years. Overall, using 2SD had the best model fit as shown by the AUC, and was associated with 21 times the hazard of participants without baseline PD-MCI. However, half of participants with baseline PD-MCI 2SD (50%) remained dementia free at six years. Previous studies have reported that having PD-MCI using Level 2 MDS criteria predicts progression to PDD over time [14, 15, 43]. The ParkWest study reported the natural history of PD-MCI to PDD over a five-year period; 39% of participants with baseline or incident PD-MCI developed PDD over five years but the majority were dementia-free (61%) [35]. Similarly, Nicoletti et al. [11] reported that the presence of PD-MCI was associated with five times the relative risk of developing PDD, although participants had a longer disease duration (mean of three years at baseline) compared to participants in the present study (mean of 5.5. months). A further study explored different applications of PD-MCI criteria [20] and found that predictive accuracy did not significantly differ between the groups. Recently, studies have investigated appropriate cut-offs for PD-MCI to identify PD participants at risk of developing PDD [21, 22]. Wood et al. [21] reported that the 1.5SD was the optimal cut-off to identify participants who developed PDD within a four-year follow-up period, with impairments in at least two tests in a single cognitive domain. However, disease duration was longer than the present study (mean symptom duration six years at baseline). In a pooled analysis of 467 participants from four cohort studies, consistent with our findings, Hoogland et al. [22] reported that applying a 1SD cut-off was not significantly associated with an increased risk of developing a PDD, but 1.5SD and 2SD cut-offs were after controlling for covariates. Moreover, they reported that 2SD was the optimal cut-off, with a hazard of over 11, which is in-keeping with our findings. However, previous studies have not explored the relative predictive value of different cut-offs of PD-MCI compared to individual neuropsychological test performance. Although PD-MCI was associated with an increased risk of PDD, we showed that impairments in specific cognitive domains had better predictive accuracy for PDD compared to PD-MCI. Therefore, using focused tests (MoCA, PoA, digit vigilance and PAL) predictive of PDD and sensitive to cognitive change has the potential to reduce large batteries of neuropsychological tests to minimise participant burden in future studies.

Our study has several strengths including using a large well-characterised cohort of newly diagnosed PD participants and an age-matched control group followed up longitudinally. We used a data-driven approach to identify neuropsychological tests sensitive to change over time and predictive of early PDD. Limitations include missing data (Supplementary Table 2) and participant attrition, which are challenging for all longitudinal studies. Participant attrition was 11–21% per time-point, and 50% cumulatively over 72 months. This is similar to attrition rates reported by previous cohorts including by Wood et al. (33% in four years) [21], by Aarsland et al. (66% in 8 years) [18] and in the CamPaIGN study (65% in 10 years) [10]. Not all participants completed tests from the CDR and CANTAB at every time point due to technical errors or a change in the protocol (54- and 72-month evaluations, Supplementary Table 2); to mitigate this, we used linear mixed effects modelling, which is able to better deal with missing data. However, a significant proportion of participants had missing CANTAB and/or CDR data at 54 months, and all had missing data at 72 months, which has implications for identifying tests sensitive to cognitive decline. Participants who declined further assessments, were lost to follow-up or died may be participants who had a faster rate of motor and cognitive decline and would have been of particular interest to this study. To mitigate this, we reviewed medical notes to ensure we identified all cases who developed PDD during the study duration.

Impaired baseline MoCA was a consistent predictor of PDD using all cut-offs, which includes tests of multiple cognitive domains; this may have implications for single-domain tests predictive of PDD. Some participants improved in their neuropsychological assessment scores which could be due to a learning effect or normal fluctuations in cognition. We used a time interval of 18 months between testing, which has been suggested as an appropriate length of time to negate practice effects; however, future studies could consider using different versions of the same neuropsychological test. Finally, this study was conceived and initiated before the PD-MCI criteria had been published by the MDS and thus our testing of visuospatial function and language was more limited than that recommended by the MDS criteria [33]. We, therefore, used a modified version of these criteria.

In conclusion, poorer performance on selective tests of global cognition, memory, verbal fluency and spatial working memory are associated with a faster rate of decline in those who develop an early PDD. PD-MCI using 2SD cut-off may be a suitable screening tool for the development of PDD within six years, but with less accuracy than impaired global cognition, attention and memory, while impaired MoCA and semantic fluency are useful screening tools for routine outpatient clinical practice. Future studies are required to understand how these cognitive deficits are associated with the pathophysiology of PDD, as well as their utility in trials of putative disease-modifying therapies.

## Supplementary Material

Supplementary Material
