# Supplementary material for: Which Neuropsychological Tests? Predicting Cognitive Decline and Dementia in Parkinson’s Disease in the ICICLE-PD Cohort
Source: J Parkinsons Dis. 2021 Aug 2;11(3):1297–308. doi: 10.3233/JPD-212581 (PMC8461722; doi:10.3233/JPD-212581)

Supplementary Material

Which Neuropsychological Tests? Predicting Cognitive Decline and Dementia in Parkinson's Disease in the ICICLE-PD Cohort

Supplementary Methods: Full Statistical Analysis

Statistical analysis was conducted using SPSS (IBM Corp. V.24, USA) and R software (Version 3.4.0; R Foundation for Statistical Computing, Vienna, Austria). Data were examined for normality of distribution with visual histograms and Kolmogorov-Smirnov's tests. Comparisons of means between two groups were performed using independent t-tests or Mann-Whitney U tests as appropriate. Ordinal data was compared using chi-squared tests. Survival and cumulative survival were calculated using Kaplan-Meier plots.

Within R, *lme4* [1] was used to perform linear mixed effects modelling (LMEM) to determine change in cognitive measures from baseline to 72 months. This form of multilevel modelling is suitable for longitudinal data analysis due to its ability to handle missing data [2], as it does not exclude subjects with missing data from the analysis. A random intercept model was used, where the intercept varied at the participant and time level. First, rate of change was modelled for all participants with group as a fixed effect, as well as interactions with time (group x time) to determine differences in rate of change of cognitive tests between Parkinson's disease (PD) participants and controls. For each cognitive test, sex, number of years of completed education, age and depression (Geriatric Depression Scale, GDS-15) were entered into the model as fixed effects. Secondly, change in cognitive scores of PD only participants was modelled, with cumulative dementia (PDD) diagnosis and interaction with time (PDD x time) included as fixed effects, to determine which tests were sensitive to change in those who developed PDD within six years. For each cognitive test, sex, number of years of completed education, age, time, disease severity (Movement Disorders Society

Unified Parkinson's Disease Rating Scale, MDS-UPDRS III x time) and depression (GDS-15) were entered into the model as fixed effects.

Backwards stepwise Cox regression identified baseline predictors of PDD using a data driven approach. Initial co-variates were: baseline age, gender, years of education, levodopa equivalent daily dose (LEDD), MDS-UPDRS III score, and GDS-15were included in the model; non-significant predictors were excluded to provide a basic model. To aid interpretation, age, years of education, LEDD and MDS-UPDRS III were dichotomised using median scores; a score of GDS-15≥10 was used to classify depression. Cognitive scores were dichotomised as impaired using: i) cut-offs at 1SD, 1.5SD and 2SD below control mean scores, and ii) using median scores (Supplementary Table 3). An additional model using impaired median scores and pen and paper only tests (Montreal Cognitive Assessment [MoCA], Mini-Mental State Examination [MMSE], semantic fluency, phonemic fluency and pentagon copying) was performed to identify tests which may be useful in a clinical setting. Impairment on each cognitive test, at the respective cut-off, was added to the basic model and a backwards step-wise Cox regression was used to identify non-significant predictors. Finally, baseline mild cognitive impairment (PD-MCI) classification using 1SD, 1.5SD and 2 SD cut-offs was also added to the basic model. Model fit was assessed using log likelihood ratios and area under the curve (AUC) was calculated for each model using receiver operating characteristic (ROC) curves. For all analysis, we applied Benjamini-Hochberg multiple comparisons correction with a 5% false discovery.

REFERENCES

[1] Bates DM, Mächler M, Bolker B, Walker S (2014) lme4: Linear mixed-effects models using Eigen and S4. *J Stat Softw.*

[2] Verbeke G, Molenberghs G, Rizopoulos D (2009) *Linear mixed models for longitudinal data*. Springer Science & Business Media.

Supplementary Table 1. Description of neuropsychological tests and measures in each cognitive domain

Cognitive domain	Neuropsychological test	Measure
Global cognition	MoCA	Total score
	MMSE	Total score
Visuospatial function	Pentagon copying from the MoCA	Modified 0 to 2 rating scale[1]
Language	Naming item from the MoCA	Number correct (0-3)
	Sentence item from the MoCA	Number correct (0-2)
Executive function	Phonemic Fluency	Number of words named
	Semantic Fluency	Number of animals named
	CANTAB: One Touch Stockings (OTS)	Number solved on first choice
Memory	CANTAB: Paired Recognition Memory (PRM)	Number correct, percentage correct
	CANTAB: Spatial Recognition Memory (SRM)	Number correct, percentage correct
	CANTAB: Paired Associated Learning (PAL)	Stages complete, total errors, total trials, mean trials to success
Attention	CDR: Simple Reaction Time (SRT)	Mean reaction time (ms)
	CDR: Choice Reaction Time (CRT)	Mean reaction time (ms), accuracy of correct responses (%)
	CDR: Digit vigilance (DV)	Mean reaction time (ms), accuracy of correct responses (%)
	CDR: Power of Attention (PoA)	Composite score of SRT, CRT and DV reaction times (ms)
	CDR: PoA reaction time variability	Coefficient of variance (CoV, %)
	CDR: Continuity of attention	Number of correct responses from CRT and DV
	CDR: Cognitive reaction time	Mean difference in reaction time between SRT and CRT (ms)
Spatial working memory (SWM)	CDR: SWM original stimuli	Mean reaction time (ms), accuracy of correct responses (%)
	CDR: SWM new stimuli	Mean reaction time (ms), accuracy of correct responses (%)
	CDR: SWM sensitivity index (SI)	Number of correct responses from SWM original and new stimuli
	CDR: SWM mean speed	Mean reaction time of SWM original and new stimuli

MoCA, Montreal Cognitive Assessment; MMSE, Mini-Mental State Examination; CANTAB, Cambridge Neuropsychological Test Automated Battery; CDR, Cognitive Drug Research.

[1]Ala TA, Hughes LF, Kyrouac GA, Ghobrial MW, Elble RJ (2001) Pentagon copying is more impaired in dementia with Lewy bodies than in Alzheimer's disease. *J Neurol Neurosurg Psychiatry* **70**, 483-488.

Supplementary Table 2. Missing cognitive data

	**Baseline (n=212)**	**18 months (n=191)**	**36 months (n=157)**	**54 months (n=128)**	**72 months (n=105)**
MoCA	Introduced later in study (n=24)	Missing data (n=1)	Missing data (n=6)	No missing data	Missing data (n=4)
MMSE	No missing data	No missing data	No missing data	Missing data (n=1)	Missing data (n=2)
CDR	Equipment failure (n=2)	Missing data (n=3)	Data collection problems (n=36)	Change in protocol (n=75)	Change in protocol (n=105)
CANTAB	Visual impairment (n=3), missing data (n=8)	Visual impairment (n=2), missing data (n=1), equipment failure (n=1)	Visual impairment (n=1), missing data (n=10)	Change in protocol (n=51)	Change in protocol (n=105)
Phonemic fluency	Missing data (n=2)	Missing data (n=1)	Missing data (n=3)	No missing data	Missing data (n=6)
Semantic fluency	Missing data (n=3)	Missing data (n=2)	Missing data (n=4)	Missing data (n=2)	Missing data (n=3)

MoCA, Montreal Cognitive Assessment; CDR, Cognitive Drug Research; CANTAB, Cambridge Neuropsychological Test Automated Battery.

Supplementary Table 3. Cut-offs of baseline neuropsychological test

Cognitive domain	Neuropsychological test	Cut-off			
		1SD	1.5SD	2SD	Median
**Global cognition**	*MoCA*	<24.5	<23.2	<22.0	<26
	MMSE	<27.9	<27.3	<26.7	<29
**Executive function**	*Phonemic Fluency*	<8.3	<6.0	<3.6	<11
	Semantic Fluency	<17.7	<14.7	<11.6	<21
	OTS number solved on first choice	<13.9	<12.6	<11.4	<15
**Memory**	*PRM number correct*	<18.2	<17.0	<15.8	<20
	PRM % correct	<76.0	<70.9	<65.8	<83.3
	SRM number correct	<71.5	<66.9	<62.3	<15
	SRM % correct	<14.3	<13.4	<12.5	<75
	PAL stages complete	<7.2	<6.9	<6.7	<7
	PAL total errors	>33.7	>41.0	>48.2	>18
	PAL total trials	>17.7	>19.6	>21.4	>14
	PAL mean trials to success	>2.4	>2.7	>2.9	>2
**Attention**	*SRT mean*	>378.3	>409.8	>441.3	>333.4
	Digit vigilance accuracy	<90.2	<87.3	<84.4	<97.8
	Digit vigilance mean	>496.8	>519.2	>541.6	>473.0
	CRT accuracy	<94.3	<92.9	<91.6	<98
	CRT Mean	>571.0	>601.1	>631.3	>524.7
	PoA	>1413.8	>1481.8	>1549.8	>1341.6
	PoA CoV	>60.4	>65.5	>70.5	>51.8
	Continuity of attention	<88.3	<86.5	<84.8	<92
	Cognitive reaction time	>248.9	>275.7	>302.5	>192.1
**Spatial working memory**	*SWM original accuracy*	<83.6	<78.6	<73.6	<100
	SWM new accuracy	<82.2	<76.2	<70.2	<100
	SWM SI	<0.7	<0.6	<0.5	<1
	SWM original speed	<1577.4	<1805.5	<2033.7	<1056
	SWM new speed	<1498.5	<1664.4	<1830.3	<1148
	SWM mean speed	<1559.3	<1762.5	<1965.7	<1117
**Visuospatial function**	*Pentagons*	<2	<2	<1	<2
**Language**	*Naming*	<3	<3	<2	<3
	Sentence	<2	<1	<1	<2

MoCA, Montreal Cognitive Assessment; MMSE, Mini-Mental State Examination; OTS, One Touch Stockings; PRM, paired recognition memory; SRM, spatial recognition memory; PAL, paired associated learning; CRT, choice reaction time; CoV, coefficient of variance; PoA, power of attention; CoV, coefficient of variance; SWM, spatial working memory

Supplementary Table 4. Neuropsychological tests modelled over time in PD vs. PDD participants controlling for baseline PD-MCI

Cognitive domain	Neuropsychological test	PD vs. PDD participants[a]					
		Time		PDD		Time x PDD	
		β	p	B	p	β	p
**Global cognition**	MoCA*	0.8	**<0.001**	-1.8	**<0.001**	-0.7	**<0.001**
	MMSE*	0.4	**0.006**	-0.4	0.075	-0.9	**<0.001**
**Executive function and Verbal fluency**	*Phonemic Fluency**	1.6	**<0.001**	-1.5	0.095	-1.1	**0.002**
	*Semantic Fluency**	0.8	0.026	-2.4	**0.016**	-1.3	**<0.001**
	OTS no. solved on first choice†	-0.7	0.911	-4.2	0.454	7.1	0.404
**Memory**	*PRM number correct†*	0.3	0.180	-0.8	0.127	-0.7	**0.003**
	PRM % correct†	1.2	0.196	-3.0	0.134	-2.9	**0.003**
	SRM number correct†	-0.2	0.278	-1.2	**0.001**	-0.2	0.472
	SRM % correct†	-1.2	0.265	-5.6	**0.001**	-0.6	0.569
	PAL stages complete†	0.0	0.675	-0.3	0.150	-0.1	0.298
	PAL total errors†	-1.5	0.293	-1.8	0.528	2.7	0.051
	PAL total trials†	-0.2	0.747	-1.3	0.115	2.0	**<0.001**
	PAL mean trials to success†	-0.1	0.434	0.1	0.412	0.3	**<0.001**
**Attention**	*SRT mean†*	-18.2	0.165	7.8	0.669	18.3	0.138
	Digit vigilance accuracy†	1.9	0.053	-3.9	0.053	-4.3	**<0.001**
	Digit vigilance mean†	-6.2	0.185	26.0	**0.005**	-0.6	0.891
	CRT accuracy†	0.3	0.361	-0.6	0.229	-0.8	**0.021**
	CRT Mean†	6.9	0.514	35.0	**0.020**	41.1	**<0.001**
	PoA†	-11.3	0.631	65.4	0.059	58.7	**0.011**
	PoA CoV†	1.0	0.406	0.5	0.771	4.1	**<0.001**
	Continuity of attention†	1.0	0.031	-2.2	**0.022**	-2.3	**<0.001**
	Cognitive reaction time†	11.7	0.234	23.9	0.128	12.0	0.192
**Spatial working memory**	*SWM original accuracy†*	-0.1	0.952	-4.5	0.083	-1.9	0.226
	SWM new accuracy†	-3.8	0.051	-0.1	0.981	-4.7	**0.015**
	SWM SI†	0.0	0.306	0.0	0.311	-0.1	0.042
	SWM original speed†	-8.9	0.913	217.2	**0.020**	256.6	**0.002**
	SWM new speed†	31.0	0.668	211.5	**0.032**	236.9	**0.001**
	SWM mean speed†	13.0	0.866	207.0	**0.023**	266.1	**0.001**

Significant results after Benjamini–Hochberg procedure are highlighted in bold p<0.023.
[a]Covariates included in the model: age, MDS-UPDRS III, Sex, GDS-15, Education, Time x MDS-UPDRS III, PD-MCI.
* Time points included: baseline, 18, 36, 54, and 72 months; †Time points included: baseline, 18, 36, and 54 months.

PD, Parkinson's disease; PDD, Parkinson's disease dementia; PD-MCI, Parkinson's disease with mild cognitive impairment using 1.5 standard deviations below normative values; MoCA, Montreal Cognitive Assessment; MMSE, Mini-Mental State Examination; OTS, One Touch Stockings; PRM, paired recognition memory; SRM, spatial recognition memory; PAL, paired associated learning; CRT, choice reaction time; CoV, coefficient of variance; PoA, power of attention; CoV, coefficient of variance; SWM, spatial working memory; MDS-UPDRS III, Movement Disorder Society Unified Parkinson's Disease Rating Scale; GDS-15, Geriatric Depression Scale.

Supplementary Table 1. CONSORT diagram of PD and control group

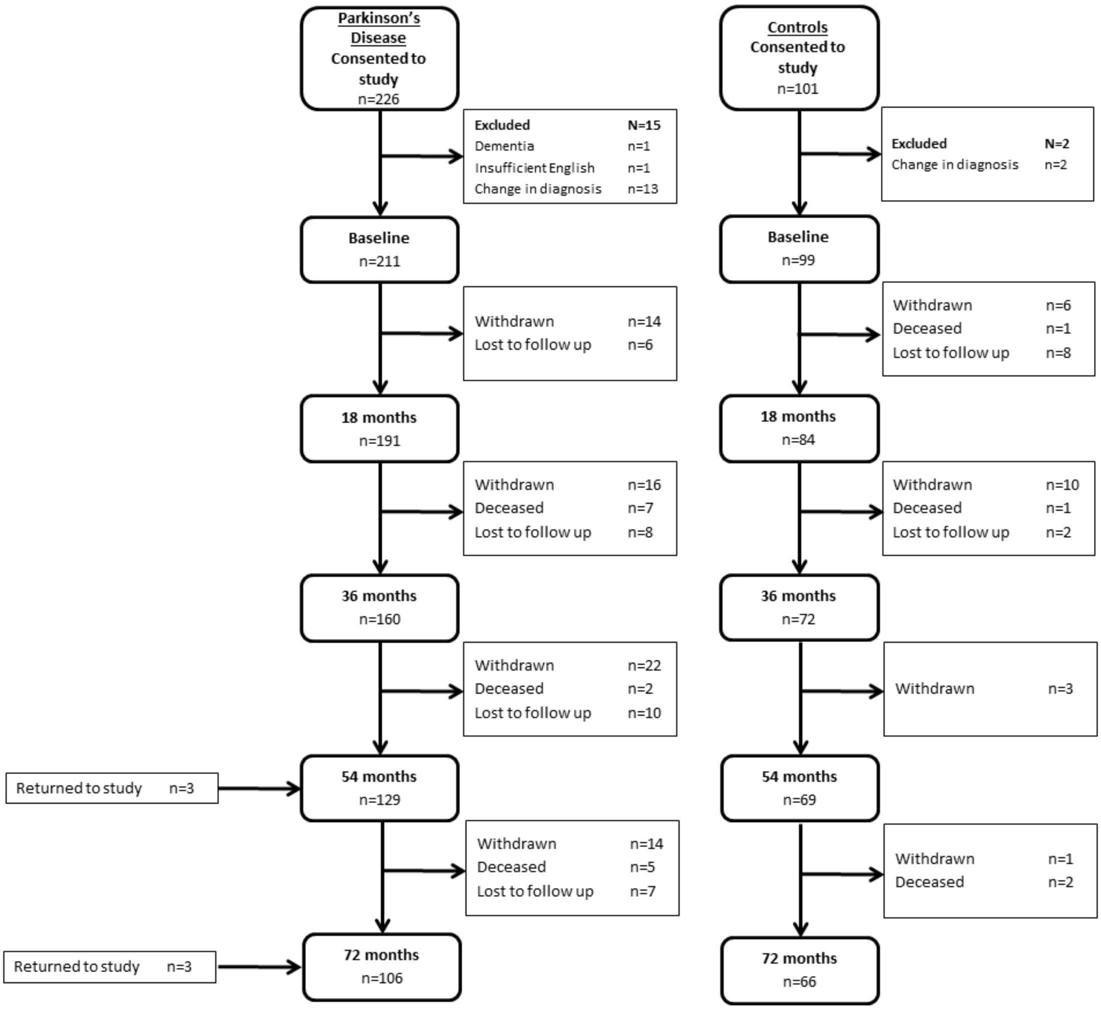

Supplement: Supplementary Material [file jpd-11-jpd212581-s001.pdf]
